# Relationship of cholinergic basal forebrain atrophy with the time course of Alzheimer's disease pathology and cognitive decline in adults with Down syndrome: a longitudinal cohort study

**DOI:** 10.1002/alz.71028

**Published:** 2026-01-11

**Authors:** Jason K. Russell, Zinayida Schlachetzki, Alexander C. Conley, Brian D. Boyd, Paul A. Newhouse, Paul S. Aisen, Michael S. Rafii, for the Alzheimer's Biomarker Consortium on Down Syndrome Study

**Affiliations:** ^1^ Alzheimer's Therapeutic Research Institute Keck School of Medicine University of Southern California San Diego California USA; ^2^ Center for Cognitive Medicine Department of Psychiatry and Behavioral Sciences Vanderbilt University Medical Center Nashville Tennessee USA

**Keywords:** acetylcholine, amyloid, AT(N), cholinergic, cognition, down syndrome, hippocampus

## Abstract

**INTRODUCTION:**

Adults with Down syndrome (DS) display increased Alzheimer's disease (AD) risk. The cholinergic system declines early in the AD continuum and relates to cognitive and functional decline. We aimed to identify the timeline of cholinergic decline in relation to hippocampal atrophy within the AT(N) framework in DS.

**METHODS:**

Three‐hundred fifty‐eight adults with DS were assessed for longitudinal changes in cholinergic basal forebrain and hippocampal volume, amyloid positron emission tomography (PET), tau PET, and cognitive performance.

**RESULTS:**

Amyloid PET increased at 36.5 years old, while tau accumulation, cholinergic basal forebrain (ChBF), and hippocampal volumetric changes occurred in the participants’ 40s. Cognitive decline on the modified cued recall test initiated at 41.7 years old. ChBF and hippocampal volumes negatively associated with AD pathology and positively associated with cognitive performance, with ChBF effects moderated by hippocampal volume.

**DISCUSSION:**

The timeline presented will inform the design of clinical trials targeting the cholinergic system or utilizing volumetric measures as biomarkers of efficacy or cognition.

**Highlights:**

- The first longitudinal assessment of cholinergic basal forebrain and hippocampal volume in DSAD.
- The AT(N) framework utilized sporadic AD is consistent in DSAD.
- Cholinergic basal forebrain volume is an alternate measure of neurodegeneration in the AT(N) framework.
- Cholinergic effects on total recall on the mCRT are moderated by the hippocampus.

## 1 BACKGROUND

Individuals with Down syndrome (DS) start accumulating amyloid early in life due to a gain of the amyloid precursor protein gene located on chromosome 21, resulting in a 95% lifetime risk of Alzheimer's dementia and making Alzheimer's disease (AD) the leading cause of death in individuals with DS.^1^ Numerous studies have assessed the progression of amyloid and tau pathology alongside cognitive decline in Down syndrome‐associated Alzheimer's disease (DSAD), demonstrating a similar, but accelerated, Alzheimer's disease (AD) time course.^2^, ^3^, ^4^ Central cholinergic decline is recognized as one of the earliest neurodegenerative changes in sporadic AD, with cholinergic degeneration occurring before hippocampal atrophy and correlating with cognitive decline.^5^

In cross‐sectional studies in DS, the cholinergic system has been demonstrated to decline as AD progresses. In DSAD, one study assessed cholinergic basal forebrain (ChBF) volumetry, describing lower volumes in more advanced AD, associating with cerebrospinal fluid measures of amyloid (amyloid β 42/40), tau (tau phosphorylated at threonine 181), and neurodegeneration (neurofilament light chain) and positive associations with hippocampal volume and cognitive performance when correcting for age.^6^ Other cross‐sectional studies revealed that cholinergic terminal density displayed a greater age‐ or amyloid‐associated reduction in adults with DS than in neurotypical controls, with positive associations between cholinergic terminal density and cognition observed.^7^, ^8^

Therapeutics targeting the cholinergic system have been extensively used in clinical practice, and novel compounds modulating the cholinergic system continue to be developed for AD.^9^ These include novel therapeutics targeting cholinergic receptors and therapeutics to preserve cholinergic function. As such, it is important to understand the timeline of cholinergic decline, including when the cholinergic system would be most sensitive to therapies or when enhancing cholinergic function versus preserving cholinergic structure is most beneficial.

This study used longitudinal and cross‐sectional data from the Alzheimer's Biomarkers Consortium–Down Syndrome (ABC–DS) cohort study to assess the population‐wide inflection points for imaging biomarkers, including ChBF volume, within the AT(N) framework, and measures of cognition. The inflection point is defined as the age at which a variable of interest (amyloid positron emission tomography [PET], tau PET, volumetric measures, and cognitive measures) is modeled to increase or decrease from a stable baseline based on longitudinal and cross‐sectional data, with ages ranging from 25 to 72 at first visit. The modeled inflection point represents, not the age of positivity or significant decline for a given measure, but rather the age at which there is a cohort level change in a variable of interest. This study investigated the temporal relationship between ChBF atrophy and established measures of the AT(N) framework, amyloid PET, tau PET, and hippocampal volume, aiming to understand the relationship between ChBF atrophy and cognitive decline. This study represents the first assessment incorporating longitudinal cholinergic measures in DSAD. These data will inform the potential utility of ChBF volumetrics in future clinical trials in DSAD, either as a cognitive biomarker or as a measure for therapeutics targeting the cholinergic system.

## 2 METHODS

### 2.1 Study design and participants

This study included adults with DS from the ABC–DS cohort study. ABC–DS is an observational, prospective, longitudinal cohort study of AD biomarkers in adults with DS, recruiting participants at 10 university sites in the United States and United Kingdom, of which seven sites were included in this analysis. The primary goal of ABC–DS is to understand the factors that moderate the relationship between Aβ, neurodegeneration, and dementia in DS, as well as identify biomarkers for those factors that could be critically important in the design of effective therapeutic trials for AD, not only in DS but also in the general population. The principal investigators and research sites of the ABC–DS study can be found at https://www.nia.nih.gov/research/abc‐ds. Data included in these analyses are from the publicly available Data Freeze 4. Eligibility for ABC–DS included age 25 years or older (with no maximum age), having trisomy 21 confirmed by karyotyping (full, translocation, or mosaic), having a premorbid mental age of at least 3 years (from standard intelligence quotient tests), and the ability to complete neuropsychological testing. Unstable psychiatric or medical conditions preventing individuals with DS from participating in the study and contraindications to magnetic resonance imaging (MRI) were exclusionary.

For inclusion in the analysis in this study, participants had to have a MRI scan that passed visual assessment without excess movement at any time point. This resulted in the exclusion of 100 visits across 79 participants (12% of available MRIs). Subset analyses were then performed on this overall group based on the availability of amyloid PET, tau PET, and cognitive data (group sizes detailed in Table 1). All participants or their legal representatives gave written consent, and all procedures were approved by a central Institutional Review Board for each study. The demographics of both cohorts are presented in Table 1.

**RESEARCH IN CONTEXT**

1. **Systematic review**: PubMed was searched from database inception to July 10, 2025. Previous studies investigated the timeline of amyloid and tau pathology and cholinergic integrity cross‐sectionally in DSAD; however, to our knowledge, no studies have assessed the cholinergic system longitudinally in the context of the full AT(N) framework in DSAD.
2. **Interpretation**: This longitudinal study of 358 adults with DS provides a timeline for cholinergic and hippocampal decline within the context of the AT(N) framework in DSAD. This timeline has implications for the design and execution of future clinical trials.
3. **Future directions**: These data suggest cholinergic basal forebrain and hippocampal volume may be useful biomarkers of cognitive performance; however, they can be complex to parcellate and may not capture early neurodegeneration. More longitudinal studies in DSAD are needed to identify functional and structural cholinergic loss not captured by MRI volumetry.

**TABLE 1:** Participant baseline demographics.

Characteristic	All participants	Amyloid subset	Tau subset	*p* value
***N***	358	273	221	
**Age (SD)**	43.1 (9.62)	42.0 (9.57)	41.3 (9.37)	0.069
**Sex (%)**				0.858
Male	203 (56.7)	161 (59.0)	129 (58.3)	
Female	155 (43.3)	112 (41.0)	92 (41.6)	
**Intellectual disability (%)**				0.757
Mild	188 (52.5)	145 (53.1)	121 (54.8)	
Moderate	141 (39.4)	101 (37.0)	77 (34.8)	
Severe	29 (8.1)	27 (9.9)	23 (10.4)	
**Race (%)**				0.998
White	346 (96.7)	265 (97.0)	217 (98.2)	
Black or African American	3 (0.8)	2 (0.7)	0 (0.0)	
Asian	4 (1.1)	3 (1.1)	2 (0.9)	
Native American	1 (0.3)	1 (0.4)	0 (0.0)	
Mixed race	4 (1.1)	2 (0.7)	2 (0.9)	
**Total visits**	686	490	364	
**Average visit number (SD)**	1.92 (0.97)	1.79 (0.73)	1.65 (0.88)	0.015

*Note*: Data are mean (SD) or *n* (%). Group differences were tested using ANOVA, chi‐squared, and Kruskal‐Wallis rank‐sum test.

Abbreviations: SD, standard deviation;

### 2.2 Procedures

Participants received neuroimaging and neuropsychological assessments every 16 months. Imaging assessments included structural T1‐weighted MRI scans, amyloid PET imaging (with [^11^C]‐PiB or [^18^F]‐Florbetapir), and tau PET imaging with [^18^F]‐Flortaucipir (FTP).

For neuropsychological measures, this manuscript focused on the modified cued recall test (mCRT) total recall score (maximum score 36), which has been previously shown to be sensitive to AD progression^10^; mCRT free recall score (maximum score 36), which was previously shown to be sensitive to early cholinergic decline^6^, ^8^; and the Down Syndrome Mental State Exam (DSMSE) (maximum score 87), which has been demonstrated to distinguish individuals with Alzheimer's dementia from cognitively stable individuals.^11^ For these assessments, a higher score indicates better cognitive performance. An assessment of premorbid intellectual disability was performed with the Stanford‐Binet Intelligence Scale, classifying participants as having mild, moderate, or severe intellectual disability.

MRI scans were parcellated using the longitudinal processing stream in FreeSurfer 7.1.^12^ MRI volumetric measures focused on the hippocampus and ChBF, two regions that have been shown to undergo atrophy early in sporadic AD^13^, ^14^ and are associated with cognitive decline in DS.^6^ The hippocampal volume was estimated using the segmentation of hippocampal subfields and nuclei of the amygdala in FreeSurfer 7.1.^15^ The ChBF volume was estimated using the ScLimbic segmentation in FreeSurfer 7.1.^16^ Cerebellar volume, segmented through the standard FreeSurfer parcellation, was compared as a region relatively unaffected by AD and total cortical gray matter was assessed as a measure of global cortical neurodegeneration. All volumes were estimated in participant‐specific longitudinal space and expressed as a percentage of estimated total intracranial volume (eTIV).

Amyloid PET was quantified using standard Centiloid conversions, as previously described.^17^ Amyloid PET images were rigidly co‐registered to T1‐weighted structural MRI scans. T1‐weighted MRI scans were transformed to Montreal Neurological Institute 152 (MNI‐152) space, and the amyloid PET scan was then transformed MNI‐152 space using the MRI transformation parameters. Once in MNI‐152 space the previously described cortical region of interest and whole cerebellar reference region were used to calculate a standard uptake value ratio (SUVR) within the cortical region of interest (ROI). These were converted to Centiloids using previously published equations.^17^, ^18^ Tau PET was quantified in the metatemporal region,^19^ a composite region specific for tau accumulation in AD. To generate SUVRs, tau PET scans were normalized on a voxel level to the inferior cerebellar gray reference region.^20^ Tau PET images were co‐registered to participant MRI scans, which were parcellated using FreeSurfer version 7.1. PETSurfer was utilized to partial volume correct the tau PET images using a Region‐Based Voxel‐wise approach.^21^, ^22^ Tau PET analysis was performed in a participant‐specific, longitudinal space derived from FreeSurfer. To harmonize MRI and tau PET across different scanners and acquisition protocols, longCombat package in R version 4.5.0 was used to separately harmonize volumetric and tau measures accounting for site and acquisition protocol, while controlling for biological covariates age and sex.^23^ To reduce variability during harmonization, scanner or acquisition protocols with fewer than 20 participants were excluded.

### 2.3 Statistical analysis

Statistical analyses were performed using R version 4.5.0. Linear mixed‐effects models, using the nlme package, with a random participant effect, controlling for sex assigned at birth and research site, were utilized to assess the relationships between imaging and cognitive measures and age. A second model was fitted with a constrained zero‐slope baseline using a piecewise linear mixed‐effects model to estimate the population‐level inflection point, where variables of interest (amyloid and tau PET, volumetric measures, and cognitive measures) begin to increase or decrease from a stable baseline, as determined by the segmented package,^24^ these models were controlled for sex assigned at birth and research site. Piecewise linear mixed‐effects models estimate inflection points based on both participant‐level longitudinal change across all ages in the dataset and cross‐sectional data points across the dataset assuming stability prior to the inflection point and linear change following the inflection point. To assess if these results replicated in just the longitudinal data, annualized rates of change of the variables of interest were assessed for 10 years prior to and 10 years following the estimated inflection point in 5‐year intervals. The annualized rate of change was calculated at the follow‐up visit, where participants had three or more visits, multiple rates of change would be calculated for that participant representing the rate of change between each visit. The mean rates of change were calculated and represented within the four 5‐year intervals to allow visualization of longitudinal change prior to and following the modeled inflection point. The coefficients of variation were calculated for hippocampal and ChBF volume before and after the inflection point to further inform their utility as biomarkers. The simple linear mixed‐effects models were compared to the constrained piecewise linear mixed‐effects models to assess which model provided the best fit. Associations between volumetric measures and PET measures of AD pathology or cognitive endpoints were assessed, correcting for age, sex, and research site, using linear mixed‐effects models. For cognitive endpoints, an interaction term between the volumetric measure and intellectual disability was included to assess differential relationships by intellectual disability. All linear mixed‐effects model relationships were represented by averaging across all covariates using the effects package in R before plotting. Finally, the participants’ baseline data, for participants with amyloid and tau PET, MRI data, and mCRT cognitive data, were z‐scored and used to generate a path analysis using the lavaan package,^25^ following the AT(N) framework to cognition with ChBF and hippocampal volume representing neurodegeneration. The path analysis was adjusted for sex assigned at birth and research site. *p* values for all analyses were false discovery rate (FDR)‐corrected for multiple comparisons. All significant estimates are reported adjusted for the covariates described, with standard error (SE) reported for estimates. Demographics across groups (MRI, amyloid subgroup, and tau subgroup) were compared using ANOVA for age, a chi‐squared test for sex and intellectual disability, Fisher's exact test for race, and the Kruskal–Wallis rank‐sum test for the number of visits per participant. AT(N) and cognitive measurements were compared across demographic (sex and premorbid intellectual disability) variables at baseline. For sex comparisons, a linear model controlling for age and research site (and intellectual disability for cognitive measures) was utilized. For comparing across premorbid intellectual disability an ANOVA adjusting for age, sex, and research site was used.

## 3 RESULTS

A total of 358 participants (203 [56.7%] male, 43.1 years old [SD = 9.62], 346 [96.7%] White, three [0.8%] Black or African American, four [1.1%] wee Asian, one [0.3%] Native American, four [1.1%] mixed race) were included in the final analysis, with an average of 1.92 (SD = 0.97, maximum 4, total 686) visits. A total of 273 and 221 participants were included in the amyloid and tau PET subgroups, respectively. There were no differences in age, intellectual disability level, study partner reported participant sex at birth, or race of the three groups; however, a significant difference was observed in average visit number (*p *= 0.015), with numerically fewer visits per participant in the amyloid and tau PET subgroups (Table 1).

Amyloid and tau levels displayed no differences based on sex or premorbid intellectual disability. ChBF volume and hippocampal volume were found to differ by sex (*p* = 0.046 and *p* = 0.0005, respectively), with males showing lower volumes in both cases. No difference in ChBF or hippocampal volume by premorbid intellectual disability was observed. Cognitive performance was found to differ by premorbid intellectual disability (mCRT total recall score, *p *= 0.0007 and DSMSE total score, *p* < 0.0001), but not by sex (Table 2).

**TABLE 2:** Baseline AT(N) and cognitive characteristics by demographics.

	Amyloid, Centiloids	Metatemporal tau SUVR	Normalized hippocampal volume	Normalized ChBF volume	mCRT total recall score	DSMSE total score
**Sex**						
Male	32.7 (40.6)	1.49 (0.63)	0.425 (0.064)	0.0418 (0.0065)	28.8 (9.07)	62.2 (13.0)
Female	25.9 (41.1)	1.44 (0.64)	0.452 (0.068)	0.0433 (0.0061)	29.6 (9.86)	62.8 (13.8)
*p* value	0.947	0.848	0.0005	0.046	0.673	0.686
**Intellectual disability**						
Mild	27.3 (39.3)	1.41 (0.63)	0.439 (0.064)	0.0425 (0.0056)	30.8 (8.4)	68.2 (11.6)
Moderate	30.1 (41.3)	1.38 (0.54)	0.436 (0.067)	0.0428 (0.0067)	28.3 (9.8)	56.6 (13.1)
Severe	17.6 (28.6)	1.28 (0.34)	0.450 (0.078)	0.0409 (0.0089)	25.7 (10.0)	45.3 (13.7)
*p* value	0.162	0.607	0.461	0.324	0.0007	<0.0001

*Note*: Data are mean (SD). Group differences were tested using linear models controlling for age, research site, and intellectual disability for cognitive comparisons (sex) or ANOVA controlling for age, sex and research site (intellectual disability).

Abbreviations: ChBF, cholinergic basal forebrain; DSMSE, Down Syndrome Mental State Exam; mCRT, modified cued recall test; SUVR, standardized uptake value ratio.

Linear mixed‐effects models controlling for sex and research site revealed a significant relationship with age for amyloid accumulation in Centiloids, tau accumulation in the metatemporal region, ChBF volume, hippocampal volume, cortical gray matter volume, cerebellar volume, DSMSE total score, DSMSE non‐memory score, mCRT total recall, and mCRT free recall (all *p *< 0.0001 after FDR‐correction Table S1). Piecewise linear mixed‐effects models with the pre‐inflection point slope constrained to zero (i.e., stable) produced an improved fit for all variables as defined by a lower Akaike information criterion (AIC) and Bayesian Information Criterion (BIC), reflected by a positive AIC and BIC differences (difference values between 13.5 to 39.7 and 8.95 to 35.2, respectively), with the exception of cerebellar volume (AIC difference: −0.361, BIC difference: −4.89) and cortical gray matter volume (AIC difference: 4.26, BIC difference: −0.266), meaning modeled inflection points for cerebellar volume and cortical gray matter volume would be unreliable (Table S2).

**FIGURE 1:**
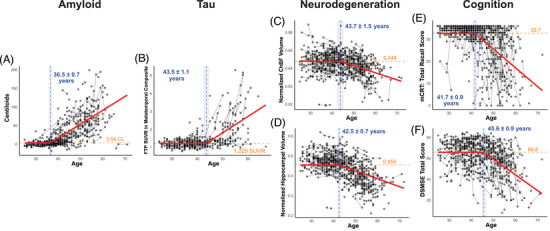
Inflection ages for biomarkers within the AT(N) framework and cognition. Shown are the modeled inflection points (in years) and baseline measures for amyloid accumulation in Centiloids (CL) (A), [^18^F]‐Flortaucipir (FTP) standard uptake value ratio (SUVR) in the metatemporal composite region (B), normalized cholinergic basal forebrain (ChBF) volume (C), normalized hippocampal volume (D), modified cued recall test (mCRT) total recall score (E), and Down Syndrome Mental State Exam (DSMSE) (F). Vertical dashed lines represent the estimated breakpoint with annotated age and standard errors, and horizontal dashed lines represent the modeled stable value prior to the breakpoint. All regression lines are modeled based on a piecewise linear mixed‐effects model with a constrained slope of zero prior to the breakpoint. Volumes are expressed as a percentage of the estimated total intracranial volume.

Amyloid accumulation in Centiloids displayed the earliest inflection point at 36.5 (SE = 0.72) years old, with an accumulation of 3.73 (SE = 0.25) Centiloids per year thereafter (Figure 1A, Table S2). The inflection point for tau accumulation in the metatemporal composite region was 43.5 (SE = 1.1) years old, with an accumulation of 0.091 (SE = 0.011) SUVR per year (Figure 1B). ChBF and hippocampal volumes started declining at 43.7 (SE = 1.47) and 42.5 (SE = 0.73) years old, respectively (Figures 1C,D). ChBF volume displayed a coefficient of variation of 13.4% before the inflection point and 10.2% after the inflection point. Hippocampal volume displayed a coefficient of variation of 9.9% before the inflection point and 11.5% after the inflection point. Cerebellar volume and cortical gray matter volume are best represented by linear relationships with age, without an inflection point (Figures S1A,B). The earliest cognitive decline was observed in the mCRT, with inflection points at 40.2 (SE = 1.2) and 41.7 (SE = 0.94) years old, for the free recall score and total recall score, respectively (Figure 1E and Figure S1). Cognitive decline, as measured by the DSMSE, occurred later at 45.6 (SE = 0.89) years old for the total score and 45.7 (SE = 1.0) for the non‐memory score (Figure 1F and Figure S1).

These modeled inflection points were visually assessed in participants with longitudinal data in the 10 years on either side of the modeled inflection point. For Centiloid values, metatemporal tau SUVR and DSMSE (total score) annualized rate of change (positive for amyloid and tau, negative for DSMSE total score) increased in 0 to 5 and 5 to 10 years following the modeled inflection point. For hippocampal volume the annualized rates of change increased (negative change) in the 0 to 5 years following the modeled inflection and for mCRT total score the annualized rate of change increased (negative change) 5 to 10 years after the inflection point. Annualized change in ChBF volume displayed no change around the modeled inflection point (Figure S2).

**FIGURE 2:**
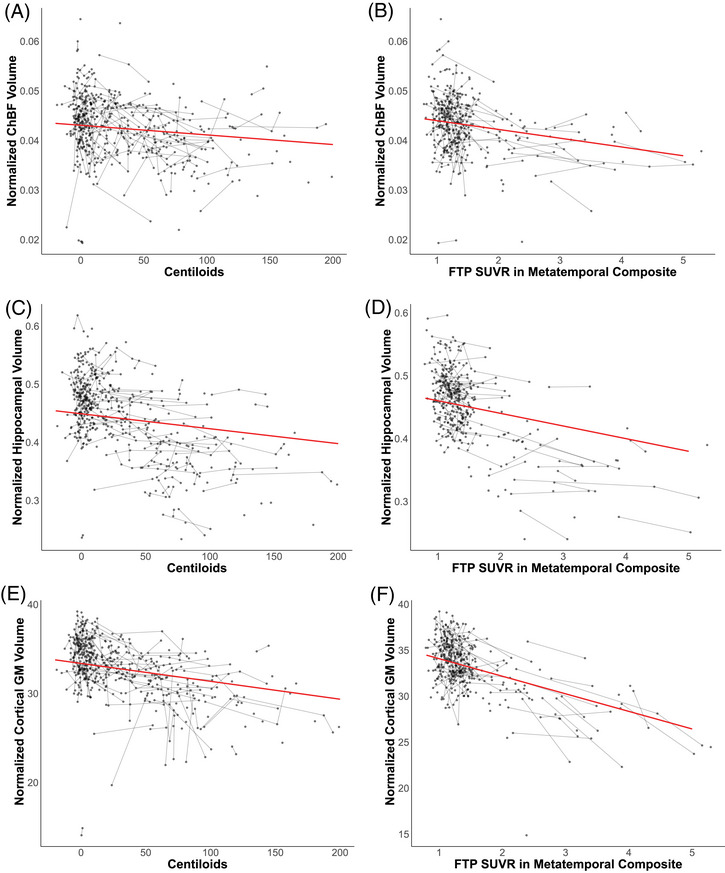
Normalized hippocampal, cholinergic basal forebrain and cortical gray matter (GM) volumes associate with PET measures of Alzheimer's disease pathology. Shown is the relationship between amyloid accumulation in Centiloids and normalized cholinergic basal forebrain (ChBF) volume (A), [^18^F]‐Flortaucipir (FTP) standard uptake value ratio (SUVR) in the metatemporal composite region and normalized ChBF (B), amyloid accumulation in Centiloids and normalized hippocampal volume (C), Flortaucipir (FTP) SUVR in the metatemporal composite region and normalized hippocampal (ChBF) volume (D), normalized cortical GM volume and amyloid accumulation in centiloids (E), and normalized cortical GM volume and FTP SUVR in the metatemporal composite region (F). All regression lines are modeled based on linear mixed‐effects models, averaged over covariates.

Increased amyloid levels measured by Centiloids were associated with reduced ChBF volume (*β* = −1.9 × 10^−5^ [SE = 9.0 × 10^−6^], *p *= 0.042 after FDR correction, Figure 2A), hippocampal volume (*β* = −2.6 × 10^−4^ [SE = 5.9 × 10^−5^], *p *< 0.0001 after FDR correction, Figure 2C), and cortical gray matter volume (*β* = −2.0 × 10^−2^ [SE = 4.3 × 10^−3^], *p* < 0.0001 after FDR correction, Figure 2E), no association with cerebellar volume was observed (Table S3). FTP SUVR in the metatemporal composite region was negatively associated with hippocampal volume (*β* = −2.0 × 10^−2^ [SE = 2.7 × 10^−3^], *p* < 0.0001 after FDR correction, Figure 2D), ChBF volume (*β* = −1.8 × 10^−3^ [SE = 4.4 × 10^−3^], *p* = 0.0001 after FDR correction, Figure 2B), and cortical gray matter volume (*β* = −1.9 [SE = 0.20], *p* < 0.0001, Figure 2F) (Table S3). Cerebellar volume displayed no relationship with FTP SUVR (Table S3).

**FIGURE 3:**
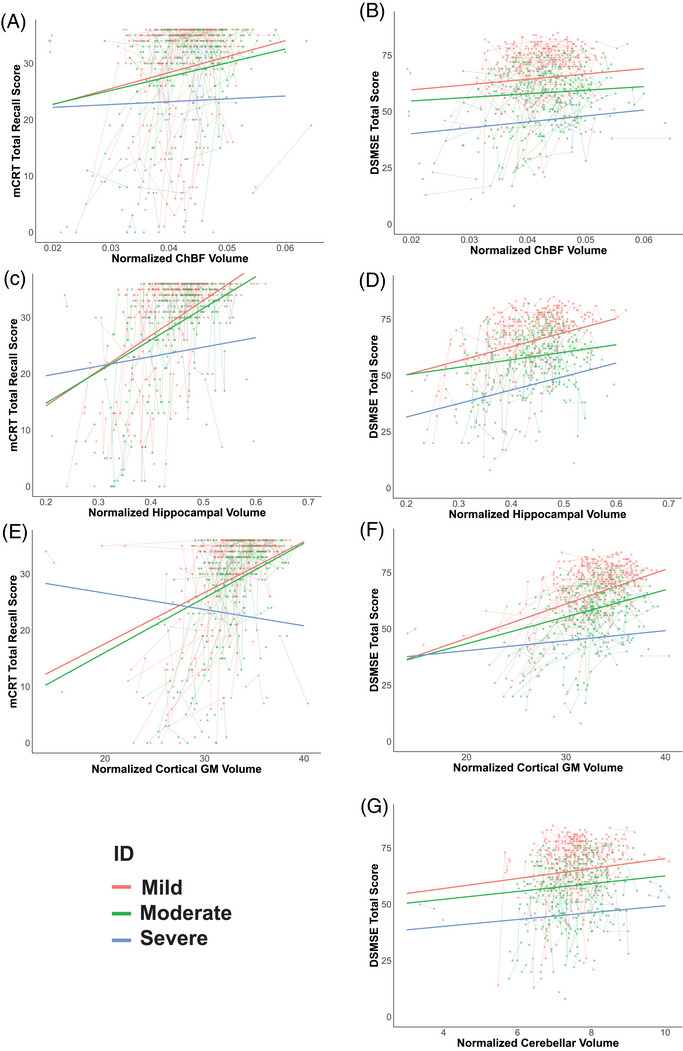
Normalized hippocampal and cholinergic basal forebrain (ChBF) volumes associate with cognitive performance. Shown is the relationship between normalized ChBF volume and modified cued recall test (mCRT) total recall score (A) and Down Syndrome Mental State Exam (DSMSE) total score (B), normalized hippocampal volume mCRT total score (C) and DSMSE total score (D), normalized cortical gray matter (GM) volume and mCRT total score (E) and DSMSE total score (F), and normalized cerebellar volume and DSMSE total score (G). All plots display different modeled fits for mild (red), moderate (green), and severe (blue) intellectual disability. All modeled fits are from linear mixed‐effects models averaged over other covariates.

Intellectual disability (mild, moderate, or severe) had no effect on ChBF (*p* = 0.324), hippocampal (*p* = 0.461), total cortical gray matter (*p* = 0.491), or cerebellar volume (*p* = 0.541) when controlling for age, sex, and experimental site (Figure S3). The mCRT total recall score was positively associated with ChBF volume (*β* = 284 [SE = 79], *p* = 0.0006 after FDR correction, Figure 3A), hippocampal volume (*β* = 62 [SE = 8.0], *p* < 0.0001 after FDR correction, Figure 3C), and cortical gray matter volume (*β* = 0.91 [SE = 0.13], *p* < 0.0001 after FDR correction, Figure 3E) (Table S4). The relationship in individuals with mild and moderate intellectual disability was similar; however, the relationship was less pronounced in individuals with severe intellectual disability, with a significant hippocampal volume × intellectual disability interaction and cortical gray matter volume × intellectual disability interaction being evident (*β* = −45 [SE = 17], *p* = 0.0095 and *β* = −1.2 [SE = 0.27], *p* < 0.0001, respectively) (Figure 3C,E, Table S4). The total score on the DSMSE was positively associated with ChBF (*β* = 235 [SE = 116], *p* = 0.049 after FDR correction, Figure 3B), hippocampal (*β* = 62.5 [SE = 12], *p* < 0.0001 after FDR correction, Figure 3D), cortical gray matter volume (*β* = 1.5 [SE = 0.2], *p* < 0.0001 after FDR correction, Figure 3F) and cerebellar volume (*β* = 2.2 [SE = 1.0], *p* = 0.033 after FDR correction, Figure 3G) (Table S4), with similar results observed for the DSMSE non‐memory score, although ChBF volume was not associated with the DSMSE non‐memory score (Table S4 and Figure S4). In the DSMSE, all levels of intellectual disability (mild, moderate, and severe) displayed similar slopes, with the exception of cortical gray matter volume where individuals with severe intellectual disability did not associate with cortical gray matter volume (*β* = 1.1 [SE = 0.36], *p* = 0.0025). All four brain regions were positively associated with the mCRT free recall score (hippocampus, *β* = 45 [SE = 6.5], *p* < 0.0001; ChBF, *β* = 216 [SE = 66], *p* = 0.0016; cortical gray matter volume, *β* = 0.69 [SE = 0.11], *p* < 0.0001 and cerebellum, *β* = 1.2 [SE = 0.56], *p* = 0.035, all after FDR correction). Positive associations were observed between the assessed brain volumes and mCRT free recall in individuals with mild and moderate intellectual disability, but not in those with severe intellectual disability (Table S4 and Figure S4).

**FIGURE 4:**
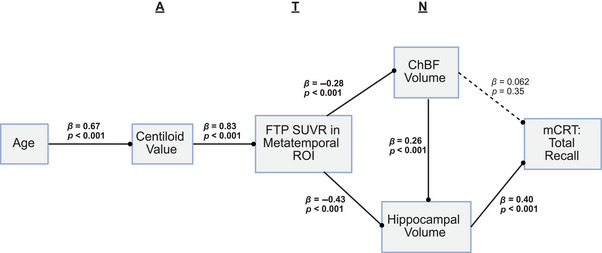
Incorporating cholinergic basal forebrain (ChBF) volume into the AT(N) framework in Down syndrome‐associated Alzheimer's disease (DSAD). Shown is a path analysis following the AT(N) framework to a final cognitive measure. Amyloid (A) is measured by amyloid PET measured in Centiloids, tau (T) is measured by [^18^F]‐Flortaucipir (FTP) standard uptake value ratio (SUVR) in the metatemporal composite region, neurodegeneration (N) is measured by normalized ChBF and normalized hippocampal volume, and cognition is measured by the modified cued recall task (mCRT) total recall score. Significant relationships are highlighted in bold with a solid connecting line, and non‐significant relationships are displayed with a dashed line.

A path analysis was performed on baseline‐only data for participants who had complete datasets across all modalities (*n* = 211). This was modeled to follow the AT(N) framework described in sporadic AD, with ChBF and hippocampal volumes representing neurodegeneration. This analysis revealed a significant path along the AT(N) framework to the mCRT total recall score as a measure of cognition. There was a direct effect of tau accumulation on ChBF (*β* = −0.28 [SE = 0.067], *p* < 0.001) and hippocampal volume (*β* = −0.43 [SE = 0.056], *p* < 0.001). While there was no direct effect of ChBF volume on mCRT total recall, there was an effect of ChBF volume that was mediated through hippocampal volume (*β* = 0.257 [SE = 0.055], *p* < 0.001) (Figure 4).

## 4 DISCUSSION

This longitudinal and cross‐sectional assessment of ChBF volume in DSAD revealed that ChBF and hippocampal atrophy initiate at a similar time, and the development of AD pathology in DSAD is consistent with the AT(N) framework described in sporadic AD. While previous studies demonstrated the temporal relationship of amyloid, tau, and cognition,^2^ the utility of the AT(N) framework in predicting AD status in DS,^26^ and the relationship between AT(N) plasma biomarkers and cognition^27^; this is the first study to incorporate longitudinal changes within the full AT(N) framework and to include ChBF atrophy as an alternate measure of neurodegeneration.

Consistent with previous reports in sporadic AD and DSAD, we observed that amyloid accumulation, measured by amyloid PET, initiated before tau accumulation and volumetric changes. Following the amyloid inflection point, the age at which accumulation initiates from a stable baseline, amyloid increased at a rate of ∼3.73 Centiloids per year. Prior studies calculated amyloid accumulation at a rate of ∼5 Centiloids/year at a Centiloid value of 30, with an increasing rate up to 100 Centiloids.^28^ The lower rate of accumulation observed is consistent with a linear model of amyloid accumulation that starts at a lower stable Centiloid level (5.15 Centiloids), when accumulation is slower, and aligns with previously described mean rate of Centiloid change from 18 Centiloids (∼3.65 Centiloids per year). The modeled pre‐inflection point baseline value of 5.15 Centiloids indicates that values exceeding 5.15 Centiloids could be one of the biological indicators of AD progression. However, due to the inherent variability associated with amyloid imaging, alternate methods will be needed to reliably detect these early changes.

In our study, tau in the metatemporal composite increased approximately 7 years after amyloid accumulation began. Previously published work in DSAD demonstrated tau increasing 2.5 to 6.1 years following amyloid positivity.^2^, ^28^ The difference between these analyses is likely due to the amyloid reference point in the present study being the inflection point from a stable baseline rather than an empirical threshold for amyloid positivity and the difference in tau ROIs assessed. Previous work assessed tau accumulation in Braak stages, whereas the present work utilized a metatemporal composite. The metatemporal composite was selected because FTP has been reported to display off‐target binding in the choroid plexus, which can affect hippocampal SUVRs (Braak stage 2), particularly alongside hippocampal atrophy.^29^ Furthermore, for clinical trial design, it has been suggested that a larger composite region, such as the metatemporal region, is more appropriate for AD‐related pathology.^19^ However, this region may fail to capture the earliest accumulation of tau in temporal and hippocampal regions, which may produce neurodegenerative changes. Alternate approaches could consider assessing tau in the ChBF; however, this would be complex due to the small volume.

Changes in hippocampal and ChBF volume occurred at approximately the same time as acceleration of tau accumulation in the metatemporal region. The timing of hippocampal decline is consistent with a recent cross‐sectional study indicating anterior hippocampal decline at 42.3 years old and posterior hippocampal decline at 40.3 years old.^30^ The first changes in cognitive function, measured by the mCRT, occurred shortly before ChBF and hippocampal volumetric changes, suggesting there may be early neurodegenerative changes not captured by volumetric analyses. Importantly, the path analysis on the cross‐sectional baseline data revealed that the AT(N) framework utilized in sporadic AD was consistent in DSAD.

The decline of the central cholinergic system has been reported as one of the earliest neurotransmitter changes in sporadic AD.^31^ Cross‐sectional studies in DS have suggested that a reduction in central cholinergic markers occurs as early AD‐related pathology develops.^6^, ^8^ In this study of DSAD, ChBF and hippocampal volume decline occurred in a similar timeframe, with ChBF volume loss occurring slightly later, although with overlapping standard errors.

To confirm that the changes in volumetry observed were specific to early AD pathology, cerebellar volume was also assessed. Cerebellar volume has been reported to decrease in sporadic AD and associate with cognitive performance, however, to a lesser extent than regions such as the hippocampus.^32^ Cerebellar volume change did not display an inflection point and was not associated with total recall score on the mCRT, a score reported to be most sensitive to early AD‐related pathology^10^; however, it did display weak positive associations with free recall score on the mCRT and the DSMSE. In contrast to ChBF and hippocampal volume, cerebellar volume did not associate with either amyloid or tau accumulation, suggesting the decline in volume observed in the ChBF and hippocampus is specific for AD pathology. Similarly, cortical gray matter volume change did not display an age‐related inflection point but did associate with both amyloid and tau pathology, most likely indicating both age‐ and AD‐related change occurring simultaneously in this region.

Better performance on the DSMSE total score was associated with larger volumes in all regions assessed. Whereas better total recall on the mCRT was associated with larger ChBF, hippocampal, and cortical gray matter volumes. The fact that hippocampal, ChBF, and cortical gray matter volumes are associated with AD pathology but cerebellar volume is not suggests that mCRT total recall is a valid measure for AD‐related cognitive change in DSAD. Interestingly, ChBF, hippocampal, and cortical gray matter volumes were positively associated with total recall on the mCRT in individuals with mild and moderate intellectual disability, but not severe intellectual disability. This suggests that the influence of intellectual disability on these cognitive measures is greater than AD‐related effects or potentially that a floor level is reached in individuals with severe intellectual disability. As such, if mCRT total recall is the primary cognitive measure for a clinical trial, caution should be exercised when recruiting individuals with severe intellectual disability. Alternatively, imaging biomarkers such as hippocampal or ChBF volumes could be particularly useful in individuals with severe intellectual disability, where standard cognitive tests are less predictive of AD‐associated neurodegenerative change. Hippocampal volume change mediated the relationship between ChBF loss and cognitive performance. This is not unexpected, given the rich cholinergic innervation of the hippocampus and the mCRT being an episodic memory task.^10^ This suggests that ChBF and hippocampal volumes are good predictors of cognitive decline, and given the consistent coefficients of variance before and after inflection points (3.2% decrease and 1.6% increase respectively), may be considered as biomarkers of cognition in future clinical trials in DSAD.

There are limitations of the current study. We assessed inflection points for different aspects of the AT(N) framework and cognition as a function of age, in contrast to previous studies that examined tau and cognition inflection points relative to amyloid chronicity.^2^ This age‐directed approach may introduce increased variability due to different ages of AD pathology progression in DS. However, assessing a single inflection point as a function of age allows for the development of age‐directed population average inflection points to inform the design of clinical trials. For example, the results of this study will enable the development of age‐related inclusion criteria and define study duration based on desired outcome measures, utilizing the inflection points and calculated rates of change. This study used constrained piecewise linear mixed‐effects models to model the inflection points and rates of change. This assumption implies stability prior to the inflection point and a constant rate of change following the inflection point, which may obscure some nuances associated with the change in these biomarkers, i.e., gradual changes prior to the modeled inflection point and variable rates of change depending on the disease state. However, for informing the design of future clinical trials, it is essential to develop a simple model that provides easily interpretable measures capturing the age of biomarker change. Additionally, for all measures, except ChBF volume, a visual analysis of a subset of participants with longitudinal data around the modeled inflection points revealed increased annualized rates of change following the modeled inflection point. Segmentation of the ChBF is complex due to its small volume. To reduce variability, we utilized a deep learning approach that allowed us to maintain the MRI scans in participant space, in contrast to other probabilistic atlas approaches that require transformation to MNI space,^33^ which may not be optimal for individuals with DS.^34^ However, this method still displays variability and may underlie the lack of a longitudinal inflection point in the subset analysis. Alternative methods such as cholinergic PET may provide a less variable, more sensitive measure of the cholinergic system and should be explored with similar longitudinal approaches.^7^, ^8^

This study utilized multimodal imaging and cognitive measures in a longitudinal study of DSAD with multiyear follow‐up. This approach enabled assessment of the AT(N) framework using measures that we believe will be directly applicable to future clinical trials. Furthermore, this is the first longitudinal assessment of cholinergic and hippocampal volume changes in DS during the development of AD‐related pathology. In this study, we observed that the progression of DSAD followed the AT(N) framework and ChBF volumetry associated with PET measures of AD pathology and cognitive performance. These data suggest that ChBF and hippocampal volume may be utilized as biomarkers for cognitive function in DSAD.

## CONFLICT OF INTEREST STATEMENT

M.S.R. is employed by the University of Southern California and the Alzheimer's Therapeutics Research Institute (ATRI) and has received grants or contracts from Eisai and Eli Lilly, which were paid to his institution. He has received consulting fees from AC Immune and Ionis. He has participated on a data safety monitoring board or an advisory board for Alzheon, Alnylam, Biohaven, Embic, Prescient Imaging, Positrigo, and Recall Therapeutics. P.S.A. has research grants from the National Institutes of Health, Eli Lilly and Eisai, and consults with Merck, Roche, BMS, Genentech, AbbVie, Biogen, ImmunoBrain Checkpoint, Arrowhead, AltPep, and Neurimmune. P.A.N. reports support from Ionis and AC Immune. The other authors declare no competing interests. Author disclosures are available in the Supporting Information.

## CONSENT STATEMENT

All participants or their legally authorized representatives for the study gave written informed consent in accordance with the Declaration of Helsinki and approved Institutional Review Board protocol.

## Supporting information



Supporting Information

Supporting Information
