# Supplementary material for: Relationship of cholinergic basal forebrain atrophy with the time course of Alzheimer's disease pathology and cognitive decline in adults with Down syndrome: a longitudinal cohort study
Source: Alzheimers Dement. 2026 Jan 11;22(1):e71028. doi: 10.1002/alz.71028 (PMC12790954; doi:10.1002/alz.71028)
Supplement: Supplementary file 1 — Supporting Information [file ALZ-22-e71028-s001.docx]

**Supplementary Material**

**Supplementary Table 1. Linear mixed-effects model relationships with age**

| **Variable** | **Slope** | **t-value** | **p-value** | **p-value: FDR Corrected** |
| --- | --- | --- | --- | --- |
| Centiloid value | 2.89 | 15.1 | 1.31 x 10^-35^ | 1.31 x 10^-34^ |
| Tau Accumulation in Metatemporal ROI | 3.98 x 10^-2^ | 8.23 | 1.06 x 10^-13^ | 1.17 x 10^-13^ |
| Basal Forebrain Volume | -2.11 x 10^-4^ | -6.51 | 2.79 x 10^-10^ | 2.79 x 10^-10^ |
| Hippocampal Volume | -2.25 x 10^-3^ | -9.53 | 3.68 x 10^-19^ | 7.36 x 10^-19^ |
| Cortical GM Volume | -2.09 x 10^-2^ | -13.3 | 1.52 x 10^-32^ | 7.60 x 10^-34^ |
| Cerebellar Volume | -2.65 x 10^-2^ | -7.86 | 5.71 x 10^-14^ | 7.14 x 10^-14^ |
| DSMSE: Total Score | -0.699 | -9.50 | 4.47 x 10^-19^ | 7.45 x 10^-19^ |
| DSMSE: Non-Memory | -0.592 | -8.34 | 2.13 x 10^-15^ | 3.04 x 10^-15^ |
| mCRT: Free Recall | -0.377 | -9.74 | 1.12 x 10^-19^ | 2.80 x 10^-19^ |
| mCRT: Total Recall | -0.520 | -10.9 | 1.21 x 10^-23^ | 4.03 x 10^-23^ |

All relationships significant. DSMSE, Down Syndrome Mental State Exam; FDR, False Discovery Rate; GM, grey matter volume; mCRT, modified Cued Recall Test; ROI, Region of Interest.

**Supplementary Table 2. Piecewise linear mixed-effects model relationships with age compared to simple linear mixed-effects models**

| **Variable** | **Inflection age** | **Standard error** | **AIC difference** | **BIC difference** | **Slope (post inflection)** | **t-value (post inflection)** |
| --- | --- | --- | --- | --- | --- | --- |
| Centiloid value | 36.5 | 0.723 | 25.8 | 21.6 | 3.73 | 15.1 |
| Tau accumulation in Metatemporal Region | 43.5 | 1.07 | 36.4 | 32.5 | 9.07 x 10^-2^ | 8.48 |
| Basal Forebrain Volume | 43.8 | 1.47 | 13.5 | 8.95 | -3.89 x 10^-4^ | -6.65 |
| Hippocampal Volume | 42.5 | 0.737 | 32.5 | 28.0 | -4.00 x 10^-3^ | -10.8 |
| Cortical GM Volume | 35.4 | 1.24 | 4.26 | -0.266 | 2.44 x 10^-3^ | -12.4 |
| Cerebellar Volume | 28.4 | 2.26 | -0.361 | -4.89 | -2.77 x 10^-2^ | -7.72 |
| DSMSE: Total Score | 45.6 | 0.891 | 39.7 | 35.2 | -1.50 | -9.85 |
| DSMSE: Non-Memory | 45.7 | 1.00 | 32.2 | 27.6 | -1.29 | -8.72 |
| mCRT: Free Recall | 40.2 | 1.19 | 29.2 | 24.7 | -0.590 | -9.70 |
| mCRT: Total Recall | 41.7 | 0.935 | 28.3 | 23.8 | -0.861 | -10.8 |

AIC, Akaike Information Criterion; BIC, Bayesian Information Criterion; GM, grey matter; mCRT, modified Cued Recall Test

**Supplementary Table 3. Linear mixed-effects model relationships between Alzheimer’s disease PET biomarkers and structural brain volumes**

| **Variable** | **Slope** | **p-value** | **p-value: FDR Corrected** |
| --- | --- | --- | --- |
| **Basal forebrain** | | | |
| Centiloid value | **-1.94 x 10^-5^** | **3.12 x 10^-2^** | **4.16 x 10^-2^** |
| FTP uptake in Metatemporal ROI | **-1.77 x 10^-3^** | **7.71 x 10^-5^** | **1.23 x 10^-4^** |
| **Hippocampus** | | | |
| Centiloid value | **-2.57 x 10^-4^** | **1.60 x 10^-5^** | **3.20 x 10^-5^** |
| FTP uptake in Metatemporal ROI | **-2.01 x 10^-2^** | **3.10 x 10^-12^** | **1.24 x 10^-11^** |
| **Cortical Grey Matter Volume** | | | |
| Centiloid value | **-2.01 x 10^-2^** | **4.98 x 10^-6^** | **1.33 x 10^-5^** |
| FTP uptake in Metatemporal ROI | **-1.91** | **<2 x 10^-16^** | **1.60 x 10^-15^** |
| **Cerebellum** | | | |
| Centiloid value | 1.62 x 10^-4^ | 0.845 | 0.845 |
| FTP uptake in Metatemporal ROI | -1.80 x 10^-2^ | 0.640 | 0.731 |

Bold values indicate significant relationships. FDR, False Discovery Rate; FTP, [^18^F]-Flortaucipir; ROI, Region of Interest.

**Supplementary Table 4. Linear mixed model relationships between structural brain volumes and cognitive performance**

| **Variable** | **Estimate** | **Volume x Moderate ID interaction p-value** | **Volume x Severe ID interaction p-value** | **p-value** | **p-value: FDR Corrected** |  |  |  |  |
| --- | --- | --- | --- | --- | --- | --- | --- | --- | --- |
| **DSMSE Total Score** | | | | | |  |  |  |  |
| Normalized Basal Forebrain Volume | **235** | 0.629 | 0.893 | **4.28 x 10^-2^** | **4.89 x 10^-2^** |  |  |  |  |
| Normalized Hippocampal Volume | **62.5** | 0.068 | 0.918 | **2.22 x 10^-7^** | **5.07 x 10^-7^** |  |  |  |  |
| Normalized Cortical GM Volume | **1.53** | 0.247 | **2.51** **x 10^-3^** | **6.76** **x 10^-15^** | **1.08 x 10^-13^** |  |  |  |  |
| Normalized Cerebellar Volume | **2.22** | 0.715 | 0.693 | **2.50 x 10^-2^** | **3.33 x 10^-2^** |  |  |  |  |
| **DSMSE Non-Memory Score** | | | | | |  |  |  |  |
| Normalized Basal Forebrain Volume | 195 | 0.663 | 0.989 | 8.23 x 10^-2^ | 8.23 x 10^-2^ |  |  |  |  |
| Normalized Hippocampal Volume | **53.5** | 0.064 | 0.837 | **4.01 x 10^-6^** | **8.02 x 10^-6^** |  |  |  |  |
| Normalized Cortical GM Volume | **1.37** | 0.116 | **1.97 x 10^-3^** | **6.30 x 10^-13^** | **3.36 x 10^-12^** |  |  |  |  |
| Normalized Cerebellar Volume | **2.15** | 0.774 | 0.634 | **2.34 x 10^-2^** | **3.33 x 10^-2^** |  |  |  |  |
| **Modified Cued Recall Test: Total Recall** | | | | | |  |  |  |  |
| Normalized Basal Forebrain Volume | **284** | 0.722 | 0.138 | **3.58 x 10^-4^** | **6.36 x 10^-4^** |  |  |  |  |
| Normalized Hippocampal Volume | **62.4** | 0.571 | **9.51 x 10^-3^** | **3.87 x 10^-14^** | **3.09 x 10^-13^** |  |  |  |  |
| Normalized Cortical GM Volume | **0.907** | 0.758 | **1.43** **x 10^-5^** | **2.36** **x 10^-11^** | **7.55** **x 10^-11^** |  |  |  |  |
| Normalized Cerebellar Volume | 1.27 | 0.580 | 0.299 | 7.11 x 10^-2^ | 7.58 x 10^-2^ |  |  |  |  |
| **Modified Cued Recall Test: Free Recall** | | | | | |  |  |  | **2.23 x 10^-7^** |
| Normalized Basal Forebrain Volume | **216** | 0.160 | **7.89 x 10^-3^** | **1.01 x 10^-3^** | **1.61 x 10^-3^** |  |  |  |  |
| Normalized Hippocampal Volume | **45.4** | 0.094 | **4.31 x 10^-3^** | **1.14 x 10^-11^** | **4.56 x 10^-11^** |  |  |  |  |
| Normalized Cortical GM Volume | **0.691** | 0.723 | **5.26** **x 10^-6^** | **1.17** **x 10^-9^** | **3.12** **x 10^-9^** |  |  |  |  |
| Normalized Cerebellar Volume | **1.24** | 0.144 | **3.34 x 10^-2^** | **2.85 x 10^-2^** | **3.51 x 10^-2^** |  |  |  |  |

Bold values indicate significant effects. DSMSE, Down Syndrome Mental State Exam; ID, Intellectual Disability; FDR, False Discovery Rate; GM, Grey Matter.

**Supplementary Figure 1. Age-relationships for cortical grey matter volume, cerebellar volume, DSMSE non-memory score, and mCRT free recall.** Shown are the linear mixed model relationships with age for cortical grey matter (GM) volume (A) normalized cerebellar volume (B) (as an inflection point did not improve fit over a linear model), and inflection point models for modified cued recall test (mCRT) free recall score (C), and Down Syndrome Mental State Exam (DSMSE) non-memory score (D). Vertical dashed lines represent the estimated breakpoint with annotated age and standard errors, and horizontal dashed lines represent the modeled stable value prior to the breakpoint. Regression lines are modeled based on a linear mixed model (A and B) or a piecewise linear mixed-effects model with a constrained slope of zero prior to the breakpoint (C and D). Volumes are expressed as a percent of the estimated total intracranial volume.

**Supplementary Figure 2. Subset of participants with longitudinal data points around the inflection point.** Shown is the annualized rate of change of amyloid in centiloids (A), tau SUVR in the metatemporal composite (B), cholinergic basal forebrain volume (C), hippocampal volume (D) modified cued recall test total score (E) and Down Syndrome Mental State Exam (DSMSE) total score (F) in 5 year intervals for the 10 years before and 10 years following the modelled inflection. Red dashed line indicates the inflection point. Bars indicate the mean annualized rate of change within the 5 year window, with error bars showing standard error of the mean. N indicates the number of data points in each time interval (each data point calculated as the annualized rate of change between two sequential visits for one participant). Additional repeated datapoints of change beyond the first for any participant are included and shown in the paranthesis (i.e. if 2 participants had 3 visits providing 2 annualized rates of change each the number in parentheses would be 2 (1 additional datapoint for each participant); if 2 participants had 3 visits and 1 participant had 4 visits the number in parentheses would be 4 (1+1+2 additional datapoints, respectively).

**Supplementary Figure 3. Intellectual disability has no relationship with the volumes of assessed brain structures.** Shown are the normalized cholinergic basal forebrain (ChBF) volume (A), normalized hippocampal volume (B), normalized cortical grey matter (GM) volume (C) and normalized cerebellar volume (D) separated by intellectual disability (mild, moderate, and severe) in adults with Down syndrome.

**Supplementary Figure 4. Brain volumes are associated with cognitive performance in adults with DS.** Shown are the significant relationships between normalized cholinergic basal forebrain (ChBF) volume (A), hippocampal volume (B), cortical grey matter (GM) volume (C) and cerebellar volume (D) and modified cued recall test (mCRT) free recall score, and relationships between normalized hippocampal (E), cortical grey matter (GM) volume (F) and cerebellar volume (G) and Down Syndrome Mental State Exam Non-memory score. All plots are grouped by intellectual disability (ID). Regression lines are linear mixed-effects models averaged across all other covariates.
